# Artificial intelligence for facial attractiveness assessment in orthodontics: model development and evaluation

**DOI:** 10.1093/ejo/cjag068

**Published:** 2026-09-18

**Authors:** Platon-Timotheos Perdikaris, Miltiadis A Makrygiannakis, Apostolos Ntokos, Eleftherios G Kaklamanos

**Affiliations:** School of Dentistry, European University Cyprus, 6 Diogenous str., Nicosia 2404, Cyprus; School of Dentistry, European University Cyprus, 6 Diogenous str., Nicosia 2404, Cyprus; School of Dentistry, National and Kapodistrian University of Athens, 2 Thivon str., Athens 11527, Greece; Independent Scholar, Athens 13123, Greece; School of Dentistry, European University Cyprus, 6 Diogenous str., Nicosia 2404, Cyprus; School of Dentistry, Aristotle University of Thessaloniki, Aristotle University of Thessaloniki Campus, Thessaloniki 54124, Greece; Hamdan Bin Mohammed College of Dental Medicine, Mohammed Bin Rashid University of Medicine and Health Sciences, Dubai Healthcare City, P.O. Box: 505055, Dubai, United Arab Emirates

**Keywords:** artificial intelligence, orthodontics, facial attractiveness, deep learning, ResNet-50, SCUT-FBP5500

## Abstract

**Background:**

Achieving valid and reproducible facial attractiveness assessment in orthodontic practice remains challenging. Artificial intelligence (AI) has shown potential in orthodontics and may help reduce inconsistencies in the evaluation of facial and smile aesthetics. This study aimed to develop and internally validate an AI model for assessing facial attractiveness in a Caucasian population.

**Materials and methods:**

The study sample was derived from a subset of the SCUT-FBP5500 V2 dataset and included Caucasian subjects, comprising 750 males and 750 females with human-rated facial attractiveness scores on a 1–5 scale. The model was based on a ResNet-50 backbone pretrained on ImageNet. Training was performed using the Adam optimizer (learning rate 0.0001) and mean squared error loss for up to 100 epochs. Stratified data splits of 70% for training, 10% for validation, and 20% for testing were created according to facial attractiveness score and sex. During training, data augmentation techniques, including resizing, random horizontal flipping, rotation, colour jittering, and tensor conversion, was applied, whereas the validation and test sets underwent resizing only. All experiments were conducted on a Linux-based system with an NVIDIA RTX 2080 GPU, using Python 3.12 and PyTorch v2.9. Model performance was assessed using MAE and RMSE across the test set, by sex, and across facial attractiveness-score quintiles.

**Results:**

The model demonstrated predictive performance on the test set, with an MAE of 0.215 (95% CI: 0.196–0.234) and an RMSE of 0.276 (95% CI: 0.251–0.300). Sex-specific analysis showed marginally better performance in males, with an MAE of 0.201 (95% CI: 0.177–0.225), compared with 0.228 (95% CI: 0.198–0.264) in females (*P* = 0.662). The highest accuracy was observed in the densely represented segment of the dataset, where the MAE was 0.156 (95% CI: 0.119–0.199). Error metrics varied across attractiveness-score ranges and were highest in the (3.02–3.63] range.

**Conclusions:**

The proposed model closely approximated human ratings of facial attractiveness and demonstrated predictive accuracy. These findings support the potential of AI-based tools to provide more consistent aesthetic assessments in orthodontics. Future research should include diverse populations to improve generalizability and clinical applicability.

## Introduction

The concept of physical attractiveness has been the subject of philosophical and scientific debate for centuries [1]. Evidence suggests that the impact of attractiveness emerges early in life. In educational settings, more attractive children are often perceived as more intelligent and socially competent, a phenomenon described as the ‘halo effect’ [2]. Within family environments, attractive children may receive more positive attention and favourable treatment from parents [3]. In adulthood, physical attractiveness continues to influence social and professional outcomes, with more attractive individuals frequently perceived as more competent, more employable, and more likely to achieve success in the workplace [4].

Facial appearance also shapes personality attributions and social perceptions. For example, a convex facial profile associated with a Class II skeletal pattern may be perceived as less dominant, whereas a more prominent chin, characteristic of Class III patterns, may be associated with increased assertiveness or aggressiveness [5]. Conversely, deviations from socially accepted aesthetic norms, including facial deformities, may contribute to teasing, bullying, and difficulties in social interaction [6, 7]. Beyond social perception, studies have linked higher attractiveness with indicators such as increased reproductive success, higher income, and even greater longevity, although these relationships are complex and influenced by multiple biological and social factors [8–10].

Research has identified several features associated with perceived facial attractiveness, including symmetry, proportional balance, averageness, youthfulness, and genetic and cultural influences [11, 12]. Many of these align with principles from art and classical aesthetics, such as the golden ratio, which has been proposed as a marker of harmonious facial proportions [13]. Skin quality and facial expression also influence attractiveness, with smoother skin and smiling faces generally rated more positively [14, 15]. Interestingly, sensitivity to facial attractiveness seems to develop early in life, as even infants have been shown to prefer faces that adults rate as more attractive [16].

In orthodontics, facial aesthetics is central to diagnosis and treatment planning. Assessment typically relies on clinical evaluation supported by cephalometric analysis and standardized photography [17, 18], with newer methods such as video analysis, stereophotogrammetry, and 3D facial scanning increasingly used in research and practice [19]. Despite these advances, aesthetic judgment remains subjective, varying with the observer’s background and assessment conditions, which can reduce agreement and reproducibility [20–24]. In addition, ratings of specific facial regions may be influenced by overall facial appearance, raising concerns about construct validity [25].

Artificial intelligence (AI), which enables computer systems to perform tasks that typically require human intelligence [26], could be useful in standardizing facial attractiveness assessment. In dentistry, AI has already been employed for several clinical tasks, including caries classification, diagnosis of cystic lesions, and assessment of alveolar bone levels [27–29]. In orthodontics, AI has also been applied to several diagnostic challenges, such as predicting skeletal growth status from lateral cephalometric radiographs [30] and identifying impacted, supernumerary, and congenitally missing teeth [31–33]. With respect to facial attractiveness, AI models have been developed to determine whether machine-based systems can predict attractiveness ratings, with the broader aim of providing a more consistent and efficient approach to facial evaluation. Several earlier studies relied on AI approaches based on landmark detection, which require a degree of human intervention [34–36]. Although these methods have shown acceptable performance, deep learning approaches have demonstrated particularly promising results [37–39].

Incorporating AI into orthodontic practice involves far more than just new software; it demands a strong understanding of the technology and supporting evidence. Clinicians must understand how models are built, tested, and validated, while also considering issues that can limit a model’s performance across diverse populations and clinical environments. Thus, before implementing any AI tool in practice, it is crucial to carefully examine the study design and reference standards, and to understand what performance metrics truly indicate. Without this knowledge, AI risks causing systematic errors and increasing disparities rather than improving diagnosis, workflow, and patient outcomes [31–33].

### Study aim

Achieving validity, reproducibility, and consistency in facial attractiveness assessment remains a challenge in orthodontic practice [20–25]. Therefore, the aim of the present study was to develop and internally validate a ResNet-50 regression model to predict crowdsourced facial attractiveness ratings in the Caucasian subset of the SCUT-FBP5500 dataset. Trained on a large, publicly available dataset, the model demonstrates a proof of concept for a computer-vision tool that could support facial attractiveness assessments in orthodontics. The model’s performance was assessed not only on the entire test set but also separately by sex and attractiveness-score ranges, highlighting where predictions are more or less reliable.

## Materials and methods

### Ethical approval and study design

All methods adhered to relevant regulations and guidelines [40]. The study used a publicly available, previously collected facial image dataset with crowd-sourced rankings and did not involve direct recruitment of participants or the acquisition of new facial photographs by the authors. Nevertheless, because facial images constitute sensitive biometric data, ethical approval was obtained from Cyprus National Bioethics Committee (2025.01.279).

### Dataset

This study used the well-established SCUT-FBP5500 dataset for assessing facial attractiveness [41]. It contains 5500 images of Asian and Caucasian individuals, including both males and females. During the creation of the dataset, 60 human evaluators (volunteers aged 18 to 27, mean age 21.6) each assigned a discrete rating of 1, 2, 3, 4, or 5 to the images. The final attractiveness score for each image was then calculated as the mean of these 60 individual ratings. Because of this averaging process, the final reference labels used for each image are continuous decimal values ranging from 1.0 to 5.0. These scores were treated as continuous reference labels for regression rather than as discrete classification categories. No formal *a priori* sample size calculation was performed because of the fixed nature of the publicly available benchmark dataset used. All eligible Caucasian facial images from SCUT-FBP5500 were included to maximise the sample size. The final sample comprised 1500 images, including 750 male and 750 female subjects, aged 15 to 60. These images are either frontal or slightly rotated. Specific demographic details regarding the exact mean age of the Caucasian subset, as well as the exact calendar years of image capture and human rating, were unavailable in the original dataset metadata.

It should be noted that the attractiveness scores in SCUT-FBP5500 represent crowd-sourced subjective human ratings rather than an objective clinical gold standard. Therefore, in the present study, these scores were used as reference labels for supervised model development, and model performance should be interpreted as the ability to approximate the dataset’s human-rated attractiveness scores rather than as an objective measurement of facial beauty.

**Figure 1:**
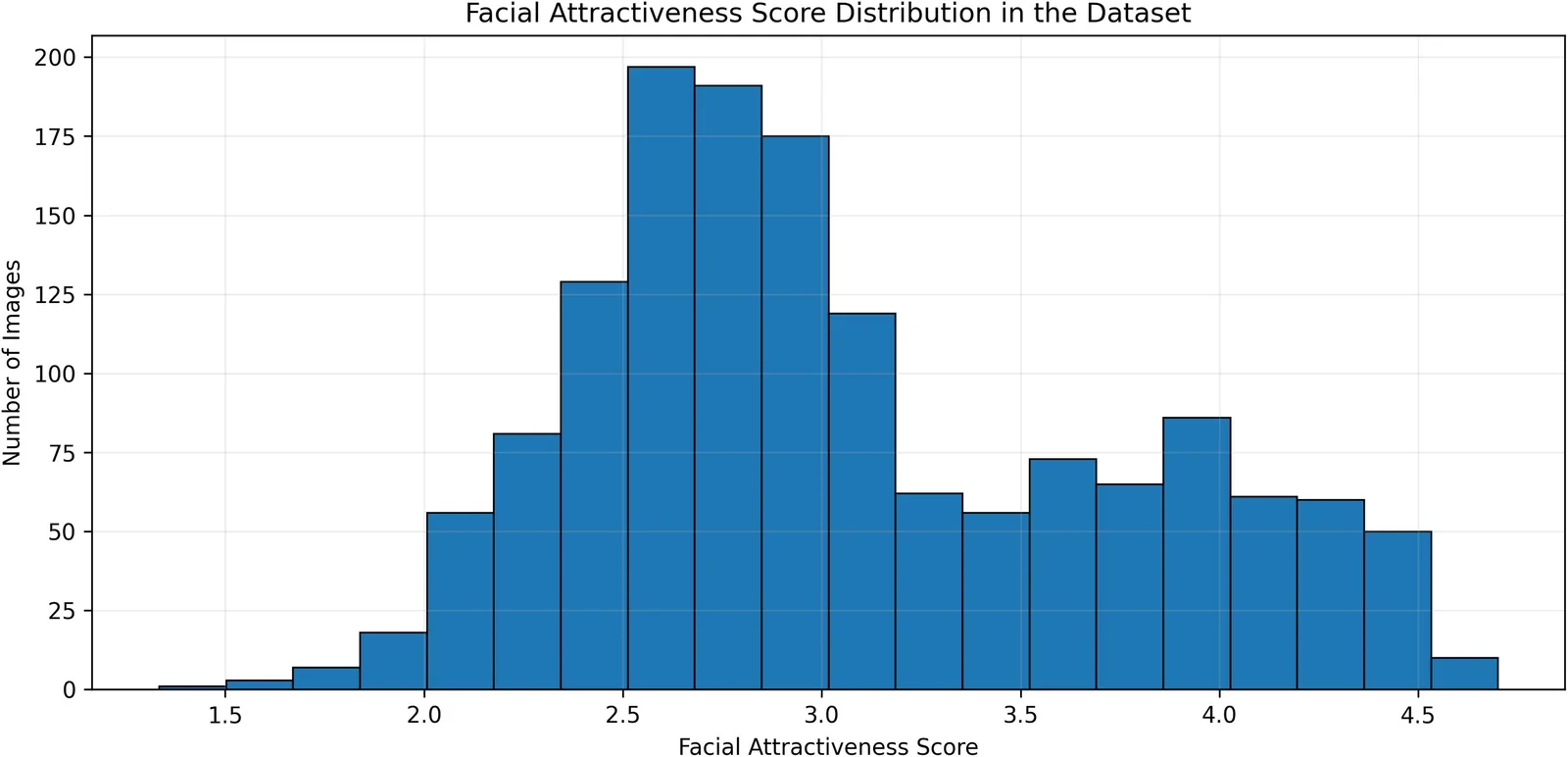
Facial attractiveness score distribution in the dataset.

Figure 1 illustrates the score distribution within the Caucasian subset. The scores were not normally distributed; instead, there was a peak around 2.6–2.8, with a smaller one between 3.75 and 4.0. This indicates that most faces received moderate-to-low ratings, while fewer were rated highly attractive. Very few images scored below 1.75 or above 4.5.

### Model architecture

The model was designed to predict the human-rated facial attractiveness score directly from each facial photograph. Unlike traditional facial analysis methods, no landmarks, cephalometric measurements, facial ratios, or manually selected anatomical features were entered into the model. Instead, the convolutional neural network (CNN) learnt relevant visual patterns automatically from the images during training. In general terms, early layers of the network identify simple image characteristics, such as edges, contours, colour transitions, and texture patterns, whereas deeper layers combine these signals into more complex facial representations. These learnt image representations were then used to estimate the attractiveness score assigned in the dataset.

A ResNet-50 CNN pretrained on ImageNet was used as the backbone model. The original classification layer was removed and replaced with a regression head so that the network produced a continuous score rather than a class label. A Squeeze-and-Excitation block was added to allow the model to automatically increase the relative importance of more informative learnt feature channels and reduce the influence of less informative channels. Importantly, this weighting was learnt from the training data and was not manually assigned to specific facial structures by the investigators. The final model output was constrained to the 1–5 range used for the reference attractiveness ratings.

Therefore, the present model should be interpreted as an end-to-end image-based prediction system. The extracted features are latent computational representations learnt by the neural network, rather than explicitly defined clinical variables. Consequently, the model does not provide direct coefficients for individual facial traits. This is a recognized characteristic of deep learning models and should be considered when interpreting their clinical applicability.

### Loss function

The model was trained with the Mean Squared Error (MSE) loss function. As a standard choice for regression tasks, MSE computes the average of the squared differences between the predicted scores and the actual ground-truth human ratings. By squaring these differences, this loss function heavily penalizes large prediction errors, effectively pushing the model toward higher accuracy without the need for manual feature weighting.

### Implementation and training details

The model was trained using the Adam [42] optimizer with a learning rate of 0.0001 for a maximum of 100 epochs. To ensure optimal generalization and prevent overfitting, we utilized an early stopping mechanism with a patience of 10 epochs, monitoring validation loss to trigger termination. The dataset was partitioned into training (70%), validation (10%), and testing (20%) subsets using stratified sampling to partly address the nonuniform distribution of attractiveness scores. This stratification was based on both the ground-truth facial attractiveness scores and subject sex, ensuring that the diverse distribution of sex and ratings was represented proportionally across all three sets to prevent data leakage. This approach was used to preserve the relative distribution of score ranges across the training, validation, and test subsets. Nevertheless, stratification could not compensate for the limited absolute number of observations in sparsely represented score ranges.

### Data augmentation and preprocessing

To prepare the images for the model, we used a series of transformations with torchvision transforms [43]. These varied between the training and test sets to promote robust learning and fair evaluation. The training pipeline included data augmentation to improve the model's ability to generalize and reduce overfitting. The steps involved resizing all images to a uniform 350 × 350 pixels, applying a 50% probability of horizontal flip to each training image to teach the model that attractiveness is not dependent on left-right orientation, randomly adjusting brightness and contrast by up to 10% to increase robustness to varying lighting conditions, applying random rotation within ± 10 degrees and converting images to PyTorch tensors by scaling pixel values from the [0, 255] range to [0.0, 1.0], making them suitable for the network. The validation and test pipeline was simpler, focusing solely on data preparation for inference rather than augmentation. Images were resized to 350 × 350 pixels to align with the training input.

### Experimental setup

All experiments were performed on a Linux-based workstation equipped with an NVIDIA GeForce RTX 2080 GPU (8 GB VRAM). The software environment was managed using Python 3.12 and PyTorch (v2.9) [44]. Data processing, statistical analysis, and visualization relied on a stack of scientific libraries, including NumPy (v2.1.3), PyTorch (v2.9), and Matplotlib/Seaborn for results plotting.

Model development and training were implemented using the PyTorch framework. To leverage hardware acceleration, computations were performed using NVIDIA’s CUDA 13.0 toolkit and cuDNN (v9.3.0) libraries, which are essential for optimizing the deep learning operations within the PyTorch backend.

### Model performance

Predictive accuracy was evaluated on the held-out test set using two primary metrics: Mean Absolute Error (MAE) and Root Mean Squared Error (RMSE).

The task was formulated as a regression rather than a classification problem. Although individual evaluators provided discrete ratings on a 1–5 scale, the reference label for each image was calculated as the aggregated average of all 60 human ratings. Because this averaging process yields a continuous value, it was most appropriate to train a regression model capable of predicting facial attractiveness as a continuous spectrum, rather than forcing the predictions into fixed, discrete categories.

MAE offers a straightforward, interpretable measure of average error, as it is expressed in the same units as the facial attractiveness scores (1–5). For instance, an MAE of 0.25 indicates that, on average, the model's predictions deviate from the ground-truth human ratings by 0.25 points.


$$\text{MAE}=\frac{1}{N}\sum_{i=1}^{N}|y_{i}-{\hat{y}}_{i}|$$

RMSE provides a complementary perspective by being more sensitive to large errors. Because the differences are squared before being averaged and square-rooted, RMSE penalizes outliers more heavily than MAE. This makes it an essential metric for identifying whether the model is prone to occasional significant miscalculations. Like MAE, RMSE is expressed in the original units of the facial attractiveness scale, allowing for direct comparison.


$$\text{RMSE}=\sqrt{}\frac{1}{N}\sum_{i=1}^{N}{\left(y_{i}-{\hat{y}}_{i}\right)}^{2}$$

### Statistical analysis

Model performance was summarized using MAE and RMSE for the entire test set, including both male and female subsets, and within specific quintiles of human-rated scores to evaluate performance across the full range of facial attractiveness. Uncertainty around the MAE and RMSE estimates was quantified using 1000 nonparametric bootstrap samples generated with replacement; 95% confidence intervals were calculated using the percentile method. Differences between male and female subjects were evaluated using two-sided Mann–Whitney U tests because normality of the prediction-error distributions was not assumed. The overall comparison was based on absolute prediction errors, whereas exploratory analyses within each attractiveness-score quintile compared signed prediction errors between sexes to assess whether the direction of prediction bias differed by sex. Statistical significance was set at *P* < 0.05. Statistical analyses were performed in Python 3.12 using NumPy v2.1.3 and the mannwhitneyu function of the SciPy library [45].

## Results

**Figure 2:**
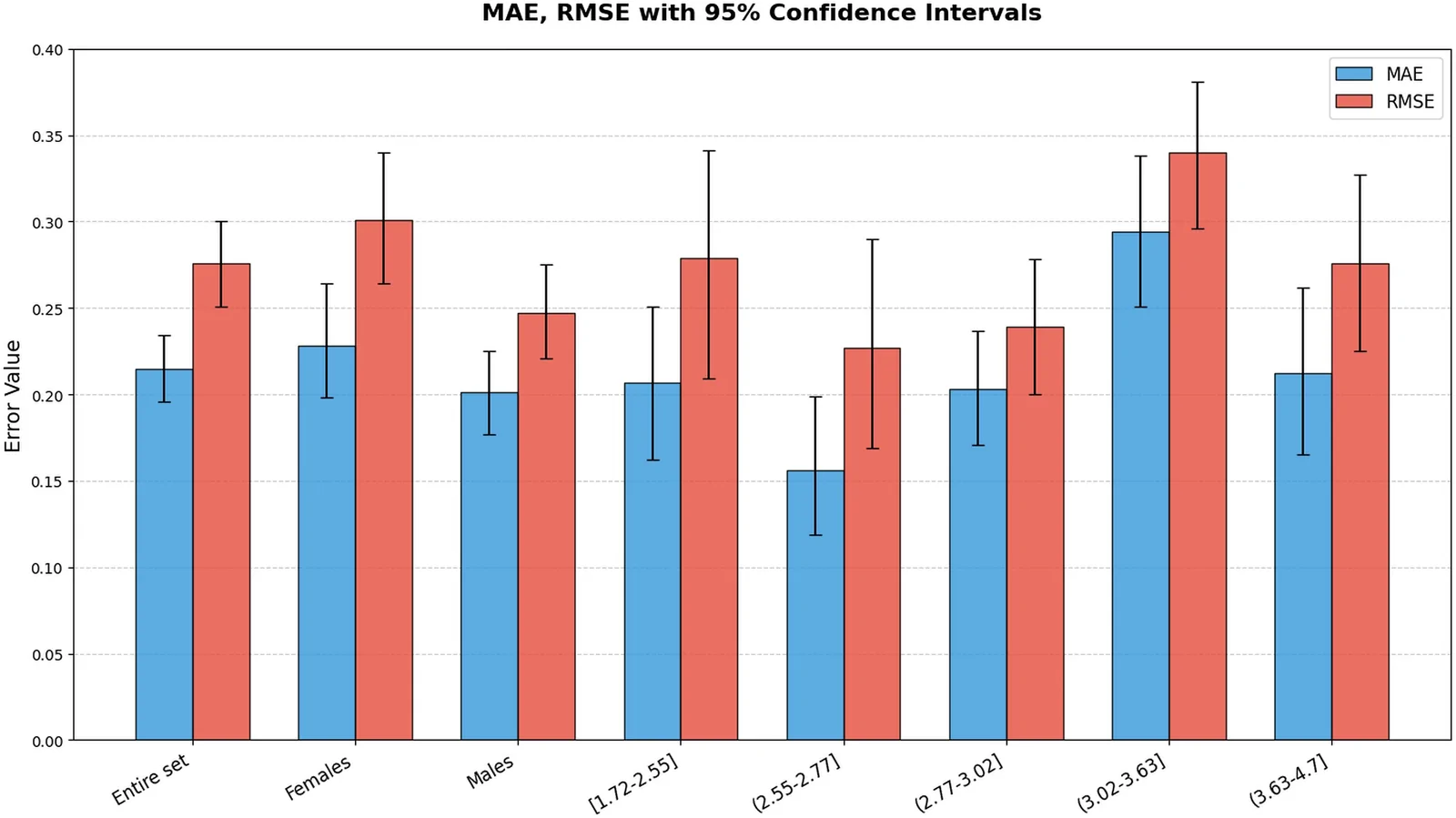
MAE and RMSE with 95% confidence intervals.

The model converged quickly, reaching its best validation performance at epoch 23. Training continued until epoch 33 without further improvement in validation loss, at which point early stopping was triggered and the weights from the best-performing epoch (epoch 23) were restored for the final model. A summary of the results across different test data subsets is shown in Table 1 and in Fig. 2.

**Table 1:** Model performance on test subsets.

Dataset	MAE (95% CI)	RMSE (95% CI)
Entire test set	0.215 (0.196, 0.234)	0.276 (0.251, 0.300)
Females test subset	0.228 (0.198, 0.264)	0.301 (0.264, 0.340)
Males test subset	0.201 (0.177, 0.225)	0.247 (0.221, 0.275)
Human-ratings score range [1.72–2.55]	0.207 (0.162, 0.251)	0.279 (0.209, 0.341)
Human-ratings score range (2.55–2.77]	0.156 (0.119, 0.199)	0.227 (0.169, 0.290)
Human-ratings score range (2.77–3.02]	0.203 (0.171, 0.237)	0.239 (0.200, 0.278)
Human-ratings score range (3.02–3.63]	0.294 (0.251, 0.338)	0.340 (0.296, 0.381)
Human-ratings score range (3.63–4.7]	0.212 (0.165, 0.262)	0.276 (0.225, 0.327)

CI, Confidence Intervals; MAE, Mean Absolute Error; RMSE, Root Mean Squared Error.

The performance metrics demonstrated strong predictive performance. The model achieved an MAE of 0.215 and an RMSE of 0.276 across the entire test set, with narrow 95% confidence intervals (CIs) indicating stable error estimation. The model achieved its highest accuracy in the human-rating range of (2.55–2.77], with an MAE of 0.156 (95% CI: 0.119–0.199), corresponding to the most densely populated segment of the dataset. Error metrics increased in less represented score ranges; notably, the (3.02–3.63] range posed the greatest challenge, with the MAE rising to 0.294 (95% CI: 0.251–0.338). This pattern was expected, as fewer training examples were available in these regions and subjective disagreement may be greater for faces rated at the lower or upper ends of the attractiveness spectrum.

The model seemed to perform marginally better on male subjects than on females. Specifically, the test subset for males had a lower MAE of 0.201 and a numerically lower RMSE of 0.247, compared with 0.228 and 0.301 for the female subset, respectively. This indicates that predictions for men are generally more accurate and consistent, with fewer large errors. However, as shown in Fig. 2, the 95% CIs for these groups overlap (Males MAE 95% CI: 0.177–0.225 vs. Females MAE 95% CI: 0.198–0.264), implying that while there is a performance difference in this sample, it may not be statistically significant. A two-sided Mann–Whitney U test confirmed this finding, revealing no statistically significant difference in absolute errors between the groups (*P* = 0.662) [45]. Therefore, there was insufficient evidence of a difference in absolute prediction errors between male and female subjects.

**Figure 3:**
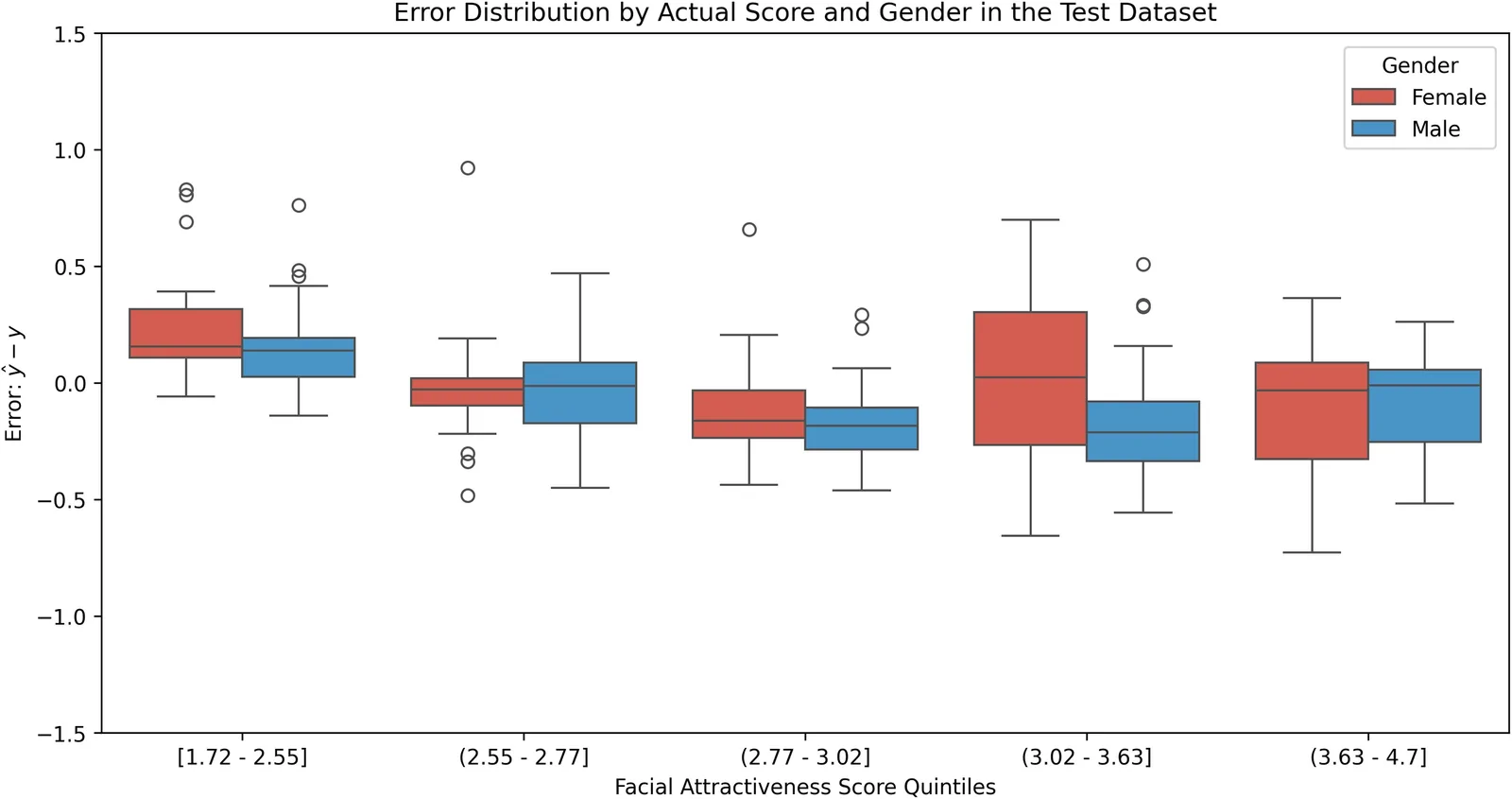
Error distribution by actual score and gender in the test dataset.

Figure 3 provides a detailed visual analysis of the model's prediction error distribution across different facial attractiveness score quintiles and sexes. A clear trend is visible where the model tends to over-predict $\left(\hat{y}\;-\;y\;>\;0\right)$ for the lowest human-rated score quintile [1.72, 2.55] and under-predict $(\hat{y}\;-\;y\;<\;0)\;$ for middle-to-high ranges, particularly between (2.77, 3.02] and (3.02, 3.63]. This behavior is common in regression tasks, where models often shift toward the more typical ‘average’ features found in the training data

The error distributions for female and male subjects were similar across most quintiles. In the lowest and highest quintiles, the median error for both sexes was close to zero or slightly positive, indicating balanced performance. Most residuals fell within ±0.5, indicating high accuracy on the 1–5 facial attractiveness scale. However, the (3.02, 3.63] quintile showed the greatest spread and error variance, likely due to increased subjectivity in ratings of ‘above-average’ attractiveness. To formally examine whether this visually observed pattern differed between sexes, a two-sided Mann-Whitney U test comparing male and female was conducted for each quintile to check for directional bias. Results showed no statistically significant differences in raw prediction errors between males and females across all five quintiles: (*P* = 0.174 for [1.72, 2.55]; *P* = 0.626 for (2.55, 2.77]; *P* = 0.428 for (2.77, 3.02]; *P* = 0.062 for (3.02, 3.63]; and *P* = 0.619 for (3.63, 4.70]). Even in the most variable (3.02, 3.63] range, sex differences were not statistically significant, despite the lowest *P*-value. These results indicate that directional bias in the model's predictions did not differ significantly between sexes across the attractiveness score quintiles.

## Discussion

### Summary of key findings

Facial assessment is essential in orthodontic diagnosis and treatment planning, combining clinical examination, standardized photographs, and cephalometric radiographs [46–48]. Despite the widespread use of these methods, challenges remain regarding consistency, reproducibility, and construct validity in facial attractiveness evaluation [20–22, 25]. The inherently subjective nature of aesthetic judgment, combined with inter-observer variability, limits the consistency of conventional approaches.

In this context, the present study aimed to develop and evaluate a ResNet-50-based AI model using publicly available crowd-sourced rankings and to contribute to the existing literature by providing a focused internal-validation analysis of AI-based facial attractiveness prediction for Caucasian male and female faces, using a public benchmark dataset with strong predictive performance. Beyond reporting overall prediction error, the study evaluated performance by sex and across attractiveness-score ranges. This analysis showed that the model performed best in the most densely represented part of the rating distribution and less consistently in sparsely represented ranges, while prediction errors did not differ significantly between male and female subjects. Thus, the study provides a computer-vision proof of concept and adds information on subgroup reliability and rating-range-dependent performance, which is important when considering the possible relevance of such tools for orthodontic facial assessment.

### Interpretation in the context of prior evidence

AI, conceptually rooted in Alan Turing’s work, enables computers to perform tasks that typically require human cognition [49, 50]. Deep learning, a key AI approach, uses multilayer neural networks to automatically extract relevant features from raw image data with minimal human intervention [51, 52]. Several deep learning architectures have been proposed [53], among which Convolutional Neural Networks (CNNs) are among the most widely used, particularly in image-based applications. CNNs can automatically detect and learn important visual features without relying on manually predefined descriptors. Their structure, loosely inspired by the organization of biological neural systems, typically consists of multiple convolutional layers followed by pooling operations, while the final layers are usually fully connected and generate the output predictions [54].

One of the principal strengths of deep learning is its capacity to integrate feature extraction and prediction within a single framework. This automated learning process contributes to the robustness and adaptability of these models and helps explain their strong performance, which, in some image-classification tasks, has approached or exceeded human-level accuracy. In addition, deep learning methods can often be transferred across tasks and data domains, a strategy commonly referred to as transfer learning [51].

In a typical deep learning workflow, the model processes the input image to identify patterns and generate a prediction through forward propagation. This prediction is then compared with the true label or rating, and the discrepancy between them is quantified as a loss value. During backpropagation, the model iteratively adjusts its internal parameters to minimize this loss, thereby improving its predictive performance over time. In medicine, deep learning has already demonstrated substantial value in diverse applications, including drug discovery [55] and cancer diagnosis [56].

In the present study, the developed ResNet-50 model demonstrated high accuracy in facial attractiveness scoring, with predictions closely aligned with human assessments. The low MAE (0.215) and RMSE (0.276) indicate a high level of predictive precision. Notably, even at the extremes of the human rating scale, the MAE remained approximately 0.21 points on a 5-point scale, supporting the model’s accuracy. Furthermore, in the rating segment with the weakest performance, the MAE remained only 0.29 points, still within an acceptable range. These findings highlight the robustness and consistency of the model and suggest that its predictive performance remained relatively low in absolute error terms across the investigated score ranges, although meaningful variation between ranges was observed.

Perceptions of facial attractiveness and the ideals that define it have evolved considerably over time [57, 58]. Throughout history, different civilizations have expressed aesthetic standards through art, particularly in sculptures and paintings. In ancient Egypt, rulers were often depicted with highly idealized facial proportions, at times exceeding realistic representation. In contrast, ancient Greek art marked a transition toward naturalism, emphasizing harmonious proportions and mathematical ratios. This focus on proportion continued in ancient Rome and persisted into the Renaissance, where facial aesthetics were guided by structured yet evolving ideals. During the Renaissance period, a typical standard of female attractiveness included features such as a rounded face, high forehead, and blonde hair [59].

In contemporary society, facial attractiveness is shaped by cultural norms that vary across populations [60]. Despite the homogenizing effects of globalization, there is increasing recognition and acceptance of individual and ethnically diverse aesthetic traits [61]. For example, within Caucasian populations, an attractive female face is often described as having a narrow facial form, rounded eyes, relatively small nose and lips, prominent cheekbones, and fair complexion [62, 63]. These standards are further influenced by external factors such as fashion trends, mass media, and the film industry, which contribute to the continuous reshaping and blending of aesthetic ideals [64, 65].

Within this evolving framework, research on facial attractiveness assessment has increasingly incorporated AI methodologies. Early approaches relied on machine learning techniques based on predefined geometric features. For instance, Iyer *et al.* [36] utilized facial landmarks to evaluate attractiveness, while Nezami *et al.* [66] developed a system that functions as a ‘facial attractiveness referee,’ assessing features based on facial ratios and angular relationships. Similarly, Fan *et al.* [67] proposed a proportional analysis model, achieving moderate predictive accuracy. More recently, Moridani *et al.* [68] employed AI methods to rank female attractiveness, reporting high levels of predictive performance.

In contrast to these approaches, deep learning models enable the direct analysis of facial attractiveness from raw pixel data, without reliance on predefined landmarks or ratios. This allows for the identification of complex, nonlinear patterns that may not be captured by traditional machine learning methods [69, 70]. Early work by Gray *et al.* [71] demonstrated the feasibility of this approach using a hierarchical feed-forward model that predicts female facial attractiveness without explicit feature extraction. More recent studies have expanded on this concept; for example, Obwegesser *et al.* investigated the influence of dental alignment and facial characteristics on perceived attractiveness using a CNN [39]. Additionally, hybrid approaches combining geometric and texture-based features have been shown to further enhance model performance [72].

At present, commercially available applications claim to evaluate facial attractiveness using AI. However, their reliability remains variable. Goshtasbi *et al.* [73] reported a significant correlation between AI-generated attractiveness scores and human ratings, although AI systems tended to assign higher scores overall. Conversely, Mamcarz *et al.* [74] found that AI-based evaluations were generally lower than participants’ self-assessments and demonstrated limited association with psychological traits. Furthermore, recent studies employing generative AI to create ‘ideal’ facial representations have revealed notable discrepancies between AI-generated outputs and classical aesthetic standards, suggesting that such systems may reflect underlying biases or evolving cultural perceptions of attractiveness [75].

Before a CNN can be effectively applied to facial attractiveness assessment, it must be trained on an appropriate and well-curated dataset to ensure robust and optimized performance. Several datasets have been developed for facial analysis research, each with distinct characteristics and applications.

One of the most widely used datasets is CelebA, introduced by Liu *et al.* [76]. This large-scale dataset comprises 202,599 images of 10,177 individuals and includes annotations for 40 binary facial attributes, such as nose size, eyebrow thickness, hair colour, and smiling expression. Derived from the CelebFaces dataset and evaluated by human raters, CelebA has become a benchmark resource in face recognition [77, 78]. In the context of facial attractiveness research, CelebA has frequently been used due to its inclusion of attractiveness labels. However, these labels are typically binary (attractive vs. unattractive), which, although simplifying model training, may limit both accuracy and fairness by oversimplifying a complex and continuous construct.

Another important dataset is FairFace, introduced by Karkkainen *et al.* [79], which consists of 108,501 facial images collected from social media platforms and online sources. Its key strength lies in its demographic diversity, encompassing seven racial groups, thereby enabling the development of models with more balanced performance across different populations and reducing algorithmic bias.

The SCUT-FBP5500 V2 dataset used in the present study is a publicly available and widely recognized resource specifically designed for facial attractiveness research. It has been extensively used in previous studies [80, 81] and offers several advantages over other datasets. Notably, it provides a relatively large and diverse collection of 5,500 facial images, including both frontal and slightly rotated views, annotated with demographic information (e.g. gender, race, and age), facial landmarks, and attractiveness scores [41]. The dataset is divided into four subgroups (Asian females, Asian males, Caucasian females, and Caucasian males), allowing for subgroup-specific analyses. Compared with binary attractiveness labels, the 1–5 scale used in SCUT-FBP5500 allows a more graded representation of perceived attractiveness. However, this scale remains relatively coarse and bounded, and therefore cannot fully represent attractiveness as a continuous perceptual construct. Compared with landmark-based or geometric-ratio approaches, the present end-to-end deep learning model did not require manual feature selection and could learn image-derived patterns directly from facial photographs. This is a methodological strength because it reduces dependence on investigator-defined aesthetic criteria. However, it also reduces interpretability, because the model cannot directly identify which anatomical traits contributed most strongly to each prediction. Finally, the sex-specific and score-range analyses provide additional information on model reliability that is often not reported in previous studies.

Furthermore, the availability of established benchmarks for architectures such as ResNet-50 enhances comparability with previous research and supports methodological consistency.

Regarding model architecture, ResNet-50, introduced by He *et al.* [69], is a state-of-the-art deep learning framework widely recognised for its accuracy and robustness in image recognition tasks. Its defining feature is the use of residual connections, which allow information to bypass certain layers, thereby facilitating the training of deeper networks and mitigating issues such as vanishing gradients. Subsequent enhancements, including Squeeze-and-Excitation (SE) modules, have further improved its performance by enabling adaptive recalibration of feature maps [82].

In addition to model architecture, data preprocessing and augmentation play a critical role in achieving optimal performance. In the present study, preprocessing techniques such as resizing and colour jittering were applied to standardize input images and enhance model generalization. Common preprocessing steps, including noise reduction, cropping, and normalization, are essential for improving data quality and training efficiency. Moreover, data augmentation strategies, such as image flipping and rotation, were employed to artificially increase the diversity of the training set, thereby reducing overfitting and improving model robustness [83].

### Strengths and limitations

Regarding the methodological strengths of the present study, several points merit emphasis. First, the model was developed using a well-established and publicly accessible dataset, which supports transparency, reproducibility, and benchmarking against prior work. By focusing on a clearly defined Caucasian subgroup with documented crowd-sourced human-rated attractiveness scores, the study was able to train and evaluate the model within a racially homogeneous reference framework. The CNN incorporated a robust ResNet-50 backbone, further enhanced with a Squeeze-and-Excitation block and an optimized regression head, and was trained according to current best practices in deep learning. The resulting model demonstrated low prediction error (MAE = 0.215) and consistent performance across both sexes and across the full range of attractiveness scores, supporting strong internal validity. In addition, the inclusion of male facial images represents an important strength, as male facial attractiveness has received less attention in the literature than female attractiveness, and its inclusion may improve the model’s relevance for broader clinical use [84].

Nevertheless, several limitations should be acknowledged. A major one is the nature of the reference standard. The SCUT-FBP5500 attractiveness scores are based on subjective human ratings and were not derived from predefined orthodontic, anthropometric, or cephalometric criteria. Although the final attractiveness score for each image was calculated as the mean of 60 individual ratings, producing continuous decimal values ranging from 1.0 to 5.0, those were based on a relatively coarse rating scale. Facial attractiveness is a culturally and ethnically influenced construct, and the demographic background of raters may affect the evaluation of faces from different populations. Therefore, although the present study focused exclusively on the Caucasian subset of the dataset, the ratings should be interpreted as crowd-sourced perceptual labels rather than a universal gold standard for Caucasian facial attractiveness.

The absence of an *a priori* sample size calculation should also be acknowledged. Because the study used a fixed public dataset, the sample size was determined by the number of eligible Caucasian images available in SCUT-FBP5500. The distribution of attractiveness scores was imbalanced, with relatively few images at the lowest and highest rating levels. This likely contributed to the increased prediction error in under-represented score ranges and limits the strength of conclusions that can be drawn for faces at the extremes of the attractiveness scale.

Moreover, because the study included only Caucasian faces aged 15 to 60 years, the applicability of the model to other racial groups, age categories, and more diverse populations remains uncertain. In addition, although the dataset reportedly consists primarily of images with neutral facial expressions, the original images were not acquired under fully standardized clinical conditions, and residual variation in pose, facial expression, lighting, hairstyle, makeup, and other appearance-related factors may have influenced both human ratings and model predictions, thereby affecting the assessment of attractiveness [15].

An additional limitation relates to the nature of the outcome itself. The present approach relies exclusively on static photographs and numerical attractiveness ratings, which do not fully capture the multidimensional nature of facial aesthetics. Perceptions of attractiveness are shaped not only by observable physical characteristics but also by subjective self-perception, cultural norms, and psychological influences [75]. Moreover, the model learnt latent image features directly from photographs. Therefore, it is not possible to state that specific anatomical features [59, 60] were individually weighted by a predefined amount. Although this end-to-end approach avoids the need for subjective manual feature selection, it also limits direct clinical interpretability. Thus, although AI can provide valuable, measurable, and more standardized information for the evaluation of facial appearance, it cannot fully account for the personal and psychosocial dimensions that are inherently involved in aesthetic judgement [74].

### Future research directions

Future studies should include larger and more balanced datasets, particularly with adequate representation of low- and high-attractiveness score categories. Moreover, research should prioritize the inclusion of diverse, multiethnic datasets encompassing a wider range of age groups, together with standardized image acquisition protocols and controlled variation in facial expressions. External validation in orthodontic patient populations is also essential to determine the model’s clinical utility and generalizability. In addition, future studies should account for factors such as makeup, accessories, and hairstyle, as these may substantially influence perceived attractiveness and, consequently, model performance [39, 63]. Moreover, explainable AI methods, such as saliency maps, Grad-CAM, occlusion testing, or feature-attribution analyses, should be incorporated to better identify which facial regions contribute most strongly to model predictions. Finally, an important gap in the current literature concerns the limited use of three-dimensional facial representations. The integration of 3D imaging into model development and training may enable more comprehensive characterization of facial morphology and, in turn, improve the accuracy and clinical relevance of attractiveness assessment [85].

### Clinical relevance

From a clinical perspective, the key implication is not that AI can objectively define facial attractiveness, but that deep learning models may approximate human perceptual ratings with measurable and testable error. Importantly, the present findings also show that model performance should not be assumed to be uniform across all facial-attractiveness levels. This supports the need for subgroup analyses, external validation, and transparent reporting before similar tools are considered for orthodontic use.

Overall, the present findings provide computer-vision proof-of-concept support for the potential applicability of AI as an adjunctive tool for facial attractiveness assessment in orthodontics. Given its strong predictive performance and close agreement with human crowd-sourced ratings, the model may contribute to more standardized and reproducible facial evaluation, supporting diagnosis, treatment planning, and patient communication. However, its use should remain clinician-guided and interpreted within the broader functional, aesthetic, and psychosocial context of care. Recent evidence suggests that AI has the potential to enhance image-based orthodontic diagnostics and treatment planning, while also highlighting important implementation challenges [86].

At the same time, the integration of AI into facial attractiveness assessment raises important ethical considerations. The use of facial images, which constitute sensitive biometric data, necessitates strict adherence to principles of informed consent, data privacy, and secure data handling. Concerns regarding transparency, accountability, and regulatory oversight have also been emphasized in the literature, particularly in the context of medico-dental facial diagnostics [87].

In addition, potential psychosocial implications should be carefully considered. Although direct evidence on AI-based attractiveness scoring remains limited, a growing body of research indicates that exposure to appearance-focused digital content may negatively affect body image, self-esteem, and overall psychological wellbeing [88, 89]. These findings suggest that the use of AI tools for attractiveness evaluation should be approached with caution, particularly in vulnerable populations, and should always be embedded within a patient-centered and ethically responsible clinical framework.

## Conclusion

The developed model demonstrated consistent performance in predicting the crowd-sourced facial attractiveness ratings available in the SCUT-FBP5500 Caucasian subset. Within the limitations of the subjective reference labels, the model provides proof-of-concept evidence that AI may support more reproducible facial aesthetic assessment. Further validation in clinically standardised, demographically diverse orthodontic populations is required before clinical implementation.

## Data Availability

The present study used the publicly available SCUT-FBP5500 dataset. No new facial image dataset was generated by the authors. The original images and attractiveness ratings are available from the SCUT-FBP5500 dataset source, subject to the terms and conditions specified by the original dataset creators. The derived study materials, including the list of included images, train/validation/test split identifiers, preprocessing details, model configuration, and analysis code, are available from the corresponding author upon reasonable request.
